# Behcet's Disease: Pakistani Experience

**DOI:** 10.12669/pjms.36.5.1916

**Published:** 2020

**Authors:** Asadullah Khan, Muhammad Haroon, Farhan Bashir, Zia ud Din

**Affiliations:** 1 Asadullah Khan, FCPS (Medicine)., Division of Rheumatology, Fatima Memorial Hospital, Lahore, Pakistan; 2 Muhammad Haroon, FRSM, FFSEM, FACR, FRCP., Division of Rheumatology, Fatima Memorial Hospital, Lahore, Pakistan; 3 Farhan Bashir, FCPS (Medicine)., Division of Rheumatology, Fatima Memorial Hospital, Lahore, Pakistan; 4 Zia ud Din, FCPS (Medicine)., Division of Rheumatology, Fatima Memorial Hospital, Lahore, Pakistan

**Keywords:** Aphthous ulcers, Behcet’s disease, Vasculitis

## Abstract

**Objective:**

To analyze the clinical manifestation of patients with Behcet’s disease, and performance of different classification criteria of Behcet’s disease in our population.

**Methods:**

It was a retrospective analysis of all Behcet’s disease patients attending Department of Rheumatology at Fatima Memorial Hospital, Lahore, Pakistan from April 2019 to July 2019. We performed a comprehensive clinical evaluation of patients with Behcet’s disease, with focus on patients’ age, gender and different clinical manifestations.

**Results:**

A consecutive cohort of 20 patients was studied. All patients met the International Criteria of Behcet’s Disease criteria, and 18 out of 20 patients also met International Study Group criteria. Mean age of the cohort was 33.5±10.4 years and 45% was female (male to female ratio of 1:1.2). Around 90% of cohort had recurrent oral and genital ulcers. Ocular involvement was present in 80% patients, while Joint manifestations were present in 75% of patients. Cutaneous, neurological (both central and peripheral nervous system involvement), and GIT symptoms were present in 50%, 30%, and 15% of patients, consecutively. Joint pain and eye symptoms were major initial symptoms in males, while eye symptoms and neurological symptoms were more common in female patients at disease onset. Reaching border line significance, Cutaneous(p-value=0.479), ocular(p-value=0.61), and GIT involvement(p-value=0.59) were more prevalent in males while neurological involvement (p-value=0.336) in females.

**Conclusion:**

Behcet’s disease occurs commonly in middle age population with equal male to female ratio, with mucocutaneus aphthosis, ocular disease and joint pains being common manifestation. Gastrointestinal symptoms are more common in males while neurological symptoms in females.

## INTRODUCTION

Behcet’s disease (BD) is a systemic variable vessel vasculitis with mucocutaneous, ocular, arthritic, vascular, neurological, cardiac, pulmonary, and gastrointestinal involvement.^1^ The disease is also known as silk road disease due to its geographical distribution along the overland trading routes from the Far East to the Europe by way of the Middle East and the Mediterranean. The prevalence of Behcet’s disease in Turkey for example is between 80 and 420 per 100,000.^2^ While it is much less in northern Europe with a prevalence of 4.9 per 100,000 in Sweden.^3^ The underlying cause of Behcet’s disease is unknown. An aberrant immune activity triggered by exposure to an agent, such as infectious, in patients with a genetic predisposition may lead to develop the disease.^4^ Common clinical manifestations include a constellation of orogenital aphthous ulcers, skin rash, ocular involvement, arthritis, gastrointestinal, arterial or venous diseases, and neurological disease. Kural-Seyahi et al. in their 2 decades-long survey found out that the “disease burden” of BD is usually confined to the early years of its course, and in many patients the syndrome ”burns out.” However, central nervous system involvement and major vessels disease are exceptions, which can have their onset late (5-10 years) during the disease course. In addition, their data showed that the disease was less severe among the females.^5^

The International Study Group (ISG) diagnostic criteria which mandates the presence of oral ulcers and addition of two more clinical features for classification are widely used with a sensitivity of 92% and specificity of 96.6%.^6^ The International Criteria for Behcet’s disease (ICBD) recently proposed and validated is an alternative criterion with 95% sensitivity and 90% specificity does not need mandatory oral ulcers presence.^7^ The relapsing and remitting nature of BD, the differences in natural course of different organ and system involvement, and the differences in the disease course between men and women, mandate that the treatment should be individualized accordingly. Untreated eye disease, vascular, nervous system and gastrointestinal system involvement can cause serious damage and even death.^8^ Topical steroid therapies, colchicine, and conventional DMARD suffice for the treatment of majority of patients. High dose pulse steroid therapy, immunosuppressants like cyclophosphamide and biologic therapy with anti TNF agents has proven efficacy in rare organ threatening and devastating complications.^9^

Local data regarding presentation of this rare disease in a Pakistani population is scarce and limited only to a few case reports.^10^,^11^ In this study, we aimed to analyze:

1. The clinical presentation of patients with Behcet’s disease.
2. The performance of different classification criteria of Behcet’s disease in our patient population.

To our knowledge, this is the largest case review series to date evaluating the clinical manifestation of Behcet’s disease among Pakistani patients.

## METHODS

It was a retrospective analysis of all BD patients attending Rheumatology Department of Fatima Memorial Hospital, Lahore, Pakistan from April 2019 to July 2019. Fatima Memorial Hospital is a tertiary care hospital and a referral center for complicated rheumatic diseases. Study was approved by ethical review committee of the institute (Ref No. FMH-04-2019-IRB-616-M, dated May 11, 2019), and informed consent was obtained from all patients. We studied the demographic data such as age and gender, clinical onset of the disease in the form of initial symptoms, duration between onset of symptoms and diagnosis of Behcet’s disease, and the fulfillment of ISG and ICBD criteria of classification of Behcet’s disease. Moreover, clinical spectrum in the form of mucocutaneous, musculoskeletal, neurological, ophthalmologic (diagnosed by ophthalmologists) and vascular disease along with the differences of these manifestations according to gender were analyzed. Patients were evaluated by a multidisciplinary team comprising of Rheumatologists, Ophthalmologist, Dermatologist and Neurologist. Ophthalmologic evaluation was performed by single ophthalmologist with slit lamp examination. Neurological diagnosis were made by a neurologist with brain and spainal cord imaging(where needed) and by clinical evaluation.

### Statistical analysis

Statistical analysis was performed using SPSS version 23.0. All the categorical variables in demographic profile and clinical manifestation were presented in form of frequency and percentage whereas Quantitative variable like age was presented in form of Mean±SD. Stratification of the data was done with respect to gender. Association between gender clinical manifestation was assessed by Chi square or Fisher’s Exact test. Mann Whitney U test was applied for the comparison of the age with respect to gender. Bar chart was used for the graphical presentation of frequency of Behcet’s disease manifestations, according to gender.

## RESULTS

Demographic data and clinical manifestation are summarized in Table-I. The mean age of the cohort was 33.5±10.4 years. Fifty-five percent of the cohort was male, and it was noted with borderline statistical significance that males were younger (median age of 31 years’ vs 36 years of females (p-value 0.119). Only 40% (n=8) of patients received the diagnosis of Behcet’s disease during their initial clinical presentation, but the diagnosis of uveitis, inflammatory arthritis, IBD and CVA was the initial diagnosis made among 35%, 10%, 5% and 5% of patients consecutively. Mean duration between first symptom and diagnosis of Behcet’s disease was 24±3 months.

**Table-I:** Demographic and clinical manifestation of patients.

Demographic and clinical manifestation	Total 20	Male 11 (55)	Female 9 (45)	P-value
Mean age (Mean±SD)	33.5±10.48	31±7.76	36±12.25	0.11
***Mucosal ulcerations***				
Oral ulcers	18	9(81.8)	9(100)	0.47
Genital ulcers	18	9(81.8)	9(100)	0.47
Acne like lesion	6(30)	4(36.4)	2(22.2)	0.64
Erythema nodosum	4(20)	3(27.3)	1(11)	0.59
Joint manifestation	15(75)	8(72)	7(77)	1.00
Arthralgia	5(25)	2(18.2)	3(33.3)	0.61
Oligoarthritis	3(15)	1(9.1)	2(22.2)	0.55
Polyarthritis	7(35)	5(45)	2(22.2)	0.40
Ocular disease	16(80)	10(91)	6(66.7)	0.61
Anterior Uveitis	5(25)	2(18.2)	3(33.3)	0.61
Posterior Uveitis	4(20)	3(27.3)	1(11.1)	0.59
Pan Uveitis	6( 30)	4(36.4)	2 (22.2)	0.64
Retinal vasculitis	2(10)	2(18.2)	0	0.47
***CNS***				
Parenchymal	3(15)	1(9)	2(22.2)	0.33
Hemispheric	2(10)	0	2(22.2)	0.55
Meningoencephalitis	1(5)	1(9)	0	
Peripheral neurologic	3(15)	1(9)	2(22.2)	0.55
Deep venous thrombosis	2(10)	2(18.1)	0	0.47
GIT involvement	3(15)	2(18.1)	1(11.1)	0.59
Gonadal involvement	3(15)	3(27.2)	0	0.21

Percentage in parenthesis, 0 = no involvement, Gonadal involvement = Testicular involvement in 3 male patients. CNS= central nervous system, Parenchymal= demyelinating lesions on brain imaging. Peripheral neurologic symptoms included mononeuritis multiplex and peripheral sensory neuropathy. GIT= Gastrointestinal tract.

In this study, 90%(n=18) patients fulfilled the ISG classification criteria, while ICBD criteria was fulfilled by all 100%(n=20) patients. Eye symptoms were the initial manifestation in 50% (n=10) patients, mucocutaneous ulcers in 40%(n=8) patients, joints pain in 25%(n=5) patients, while neurological deficit in 10% (n=2) patients (Table-II).

**Table-II:** Initial most symptoms according to gender.

Symptom	Male	Female	P-value
Joint pain	4 (36.4)	1 (11.1)	0.31
Eye problems	6(54.5)	4 (44.4)	1.00
Apthus ulcers	1 (9.1)	1 (11.1)	1.00
Neurological	0 (0)	2 (22.2)	0.18

Percentage in parenthesis, 0 = not as initial manifestation.

Over all, most prevalent clinical symptom was aphthosis (oral, genital aphthous ulcers) which was present in 90% (n=18) patients. Ocular disease was present in 60% (n=12) patients manifesting as pan-uveitis in 30%(n=6), anterior uveitis in 25%(n=5), posterior uveitis in 20%(n=4), and retinal vasculitis in 10%(n=2). Cutaneous symptoms consisted of acne like lesions in 30%(n=6) and erythema nodosum (EN) like lesion in 20%(n=4). Neurological insult was noted in 30%(n=6) patients, and included cerebrovascular accident (CVA) in 10%(n=2), meningoencephalitis in 5%(n=1), peripheral sensory neuropathy (diagnosed clinically by a neurologist) in 10%(n=2) and mononeuritis multiplex in 5%(n=1) of patients. Deep venous thrombosis as the only vascular involvement was present in 10%(n=2) patients.

Although not statistically significant, joint pain and ocular disease were more common initial presentation in males, while ocular manifestation and neurological involvement was more common initial manifestation in females (Table-II). Male patients had preponderance of cutaneous, ocular, vascular and GIT involvement, while female patients had more ocular and neurological involvement than male patients (Table-III). Joint in females were oligoarthritis and arthralgias while Polyarthritis was more common in males; ocular diseases were more in form of anterior uveitis in females while posterior uveitis and pan-uveitis in males; neurological manifestation in females were in form of hemiparesis, in addition, peripheral sensory neuropathies were more common in females. Vascular disease in the form of DVT was confined to male patients.

**Table-III:** Different clinical system involvement according to gender.

Clinical System	Male	Female	Odds ratio	Confidence Interval	P-values
Cutaneous manifestation	4 (36.4)	2 (22.2)	2.4	0.387-14.881	0.40
Joint disease	2 (18.2)	3 (33.3)	0.76	0.097-5.958	1.00
Ocular disease	10 (91)	6 (66.7)	2.25	0.285-17.759	0.61
Neurological diasese	1(9)	2 (22.2)	0.28	0.037-2.092	0.33
GIT involvement	2 (18.1)	1 (11.1)	2.4	0.255-35.334	0.59

Percentage in Parenthesis, CI=Confidence interval, CNS=central nervous system, GIT=gastrointestinal tract.

## DISCUSSION

Behcet’s disease vary in its clinical presentation across the globe. We demonstrated the orogenital aphthosis, articular symptoms, and ocular disease to be the most prevalent manifestation in our cohort. There was paucity of vascular involvement in our findings. Both diagnostic criteria had excellent sensitivity in the cohort. This is the largest study of clinical picture of Behcet’s disease in the country to the best of our knowledge. The cohort however is not a true representative of the country’s population as most of the patients were referred to the tertiary care referral center.

This study is clinically important for a number of reasons. Firstly, we studied the most common clinical presentation of BD in our population. On comparison with cohorts from other regions, the authors found a higher prevalence of oral ulcers (100% vs 89%), genital ulcers (90% vs 77%), and ocular involvement (80% vs 43%) in comparison to Pande et al. who presented the clinical manifestation from a neighboring country.^12^ The probable reason was the inclusion of 27% of patients with diagnosis of incomplete Behcet’s with incomplete clinical spectrum in their cohort; however, we included only the patients with complete diagnostic features of Behcet’s disease. Average duration of symptoms to diagnosis was lesser in our study (24.3 months’ vs 53 months) which can be due to increased and earlier referrals to rheumatology over time.^13^ In comparison to cohorts from Turkey and USA^14^, the cohort showed higher prevalence of ocular involvement (80% vs 40% USA and 34% Turkey), and genital ulcers (90% vs 78% in USA and 80% Turkey). The prevalence was nearly equal in three populations in mean age of patients, prevalence of oral ulcers, joints, and vascular involvement, while our study population showed lower prevalence of neurological and gastrointestinal insult. The possible causes of such variations can be multifactorial such as ethnicity and geographic background, but these will require further evaluation. El-Garf et al.^15^ studying the Egyptian population found similar results of prevalence of mucutaneous ulcers, skin disease, neurological and ocular diasese, however pan-uveitis was predominant eye lesion in our study (30% vs 10%) and vascular insult in our study was significantly low (10% vs 59%).

**Fig 1:**
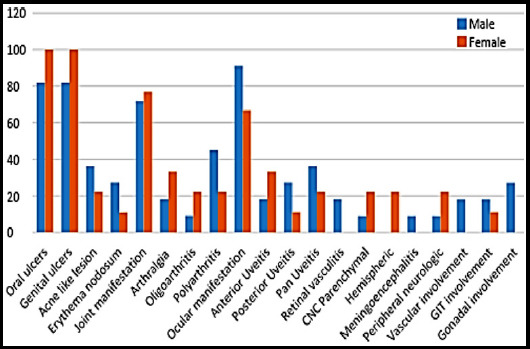
The frequency of Behcet’s disease manifestations, according to gender.

Secondly, We evaluated the initial presenting complaint of patients in the cohort. Eye problem was the initial-most symptoms in 50% of our patients (54% male vs 44% female, followed by joint symptoms in 25 % (36% male’s vs 11% females), while neurological symptoms were the first symptoms in 22.2% females only. This is in contrast to findings of other cohorts which showed mucocutaneous symptoms as most initial presenting symptom of the disease.^16^,^17^

Thirdly, we studied the gender differences in clinical spectrum of BD. There was a slight male predominance of 1.2:1 reflecting other Asian countries like Turkey and middle Eastern countries, while Western countries, and far Eastern countries like Korea show more female predominance.^18^-^20^ Among mucocutaneous symptoms, genital ulcers were slightly higher in female population as was observed by Tursen et al.^21^ Skin lesions like EN and acne were more common in male as compare to female, this is in contrast to Tursen et al., and Yazici et al., who reported EN and acne like lesions to be more common in female.^21^,^22^ Among other systemic features, our cohort had statistically insignificant male predominance in ocular (p-value 0.61), vascular (p-value 0.47), gonadal (p value 0.218) and GIT involvment (P-value 0.59); and a female predominance in neurological insult (p-value 0.336). The findings are concordant with Tursen et al. in male predominance of ocular and vascular system insult but discordant to neurologic involvement which was more prevalent in male patients in their study.^21^

Fourthly, we evaluated both diagnostic criteria of BD in our cohort. Hundred percent of patients fulfilling ICBD criteria and 95% fulfilling ISG criteria reassures the use of the two criterias for diagnosis of BD in our population. Such high sensitivities of these criteria in our cohart could be due to fulfillment of collective criteria instead of simultaneous presence of all symptoms; however, a bigger sample size would elaborate the sensitivity in our population further.

Peculiar findings of the study included the pattern of joint disease and initial most presenting symptoms. The study showed 35% of joint disease manifesting as polyarthritis, this is in contrast to higher prevalence of monoarthritis and oligoarthritis pattern.^23^

We demonstrated a delay of 24±3 months from initial symptoms to diagnosis of the syndrome. The possible causes include delay in referral to rheumatologist, incomplete clinical picture of disease at presentation, atypical initial presentation of Behcet’s as mentioned above as compared to the common understanding of aphthosis as the initial most presentation.

### Limitations of the study

The study should be evaluated in light of some limitations in the form of small sample size, single center study, retrospective analysis and lack of inclusion of treatment or disease activity. There is a risk of selection bias since this was not a population-based study. We do recommend above mentioned points to be the possible scope of future research in our population.

## CONCLUSION

Behcet’s disease manifests as a multisystem inflammatory disease of young to middle age population. Ocular, orogenital aphthosis, and musculoskeletal symptoms are common to both genders; GIT and vascular disease are predominantly in male patients and neurological insults mainly in female patients. Both ISG and ISBD classification criteria has excellent sensitivity for its diagnosis in our study.

### Author’s Contribution

**AK** Conceived the idea, designed the study, data collection, statistical analysis, manuscript writing and is responsinble for integrity of research.

**MH** Data analysis, and manuscript writing, expert review, final approval of manuscript.

**FB and ZD** Data collection, data analysis.
